# Mobile addiction treatment units: a narrative review

**DOI:** 10.1186/s13722-025-00619-1

**Published:** 2025-12-24

**Authors:** Irving Barrera, Grace Wang, Brammy Rajakumar, Siva Muthupalaniappan, Alexandria E. Cronin, Avik Chatterjee

**Affiliations:** 1 Harvard Medical School, Boston, MA USA; 2 Countway Medical Library, Harvard University, Cambridge, MA USA; 3 Section of General Internal Medicine, Boston Medical Center, Boston University Chobanian & Avedisian School of Medicine, Boston, MA USA; 4 Grayken Center for Addiction, Boston, MA USA

## Abstract

**Background:**

Drug overdose deaths have increased in the last decade, becoming a substantial public health priority. Mobile Addiction Treatment Units (MATU) are vans, vehicles, or portable clinics that provide low-threshold, low-barrier, community-based services for addiction treatment including opioid agonist medications. MATUs are a point of entry for care, particularly for individuals who have faced barriers to access at in-person healthcare facilities.

**Objective:**

This narrative review aims to synthesize and conduct a thematic analysis of the research on implementation and outcomes of MATUs in the United States. Our study’s primary objectives were threefold: 1) to evaluate MATU program reach, 2) to evaluate MATU program effectiveness, and 3) to evaluate MATU program implementation.

**Methods:**

We identified studies examining MATUs by searching electronic databases MEDLINE (Ovid), Embase (Elsevier), PsycINFO (EBSCO), and Web of Science Core Collection (Clarivate). Records were selected for full-text review if their abstract referenced any mobile modality for substance use treatments. Exclusion criteria included review articles, opinion articles, theoretical articles, abstracts, dissertations, studies conducted outside of the United States, and studies focused solely on mobile harm-reduction interventions without offering medication treatment for SUD. This review is reported per the Preferred Reporting Items of Systematic Reviews and Meta-Analyses for Scoping Review (PRISMA-ScR) guidelines.

**Results:**

A total of 2,232 articles were screened at the title and abstract level, of which 83 were assessed for full text eligibility. The 34 articles selected for inclusion were varied in methodology, and included randomized controlled trials (RCTs), observational studies, cohort studies, and mixed-methods research. The most common study locations were Baltimore, MD (10 studies), Boston, MA (5 studies), Philadelphia, PA (4 studies), and New Haven, CT (3 studies). Regarding reach, four studies were conducted during the COVID-19 pandemic. Six studies were conducted primarily in a population experiencing homelessness; two studies were conducted primarily in populations with criminal justice involvement; four studies were conducted primarily in youth or young-adult populations; three studies were conducted in rural populations. In these settings, MATUs successfully engaged vulnerable and underserved populations, delivering comprehensive care that combined harm reduction, primary care, and mental health services. These units demonstrated potential to enhance health outcomes, reduce stigma, increase treatment retention rates in marginalized populations compared to office-based programs, and tackle social determinants of health. Common challenges included patient engagement, logistical and regulatory barriers, and financial sustainability, all compounded by limited space, staffing, and resources, while homelessness, encampment removals, and the COVID-19 pandemic further disrupted care continuity (J Subst Abuse Treat 120:108149, 2021; Front Public Health 11:1154813, 2023; J Subst Use Addict Treat 159:209272, 2024; J Subst Use Addict Treat 164, 2024; Health Place 28:153–66, 2014; Addict Sci Clin Pract 18:71, 2023).

**Conclusion:**

MATUs proved to be innovative and effective in addressing OUD and related issues for vulnerable populations traditionally lacking access to care. However, ongoing efforts to overcome implementation challenges and ensure sustainable funding and resources are crucial for their continued success and expansion. Future research should focus on large-scale, quantitative studies, particularly in diverse and rural settings, to better understand their long-term impact and sustainability.

**Supplementary information:**

The online version contains supplementary material available at 10.1186/s13722-025-00619-1.

## Introduction

Over the last two decades, drug overdose has been a leading cause of death in the United States. From 2012 to 2022, drug overdose deaths increased from 41,502 to 109,413, with opioids accounting for more than 50% of these deaths [1]. Although 2024 showed a decrease of 27% in drug overdose deaths, drug overdoses remain a public health priority [2].

Although Medications for Opioid Use Disorder (MOUD) are associated with substantially lower risk of death, only a minority of individuals at risk receive MOUD prescriptions [3]. MOUD are often prescribed in outpatient settings, which can be inaccessible due to strict induction protocols and program requirements, stigma, and deficiencies in clinical support [4]. Overdose rates are historically high among marginalized populations, especially people experiencing homelessness [5, 6]. Yet individuals experiencing homelessness are substantially less likely to be prescribed MOUD than their housed counterparts [7]. Marginalized individuals with substance use disorders experience significant and compounding barriers to treatment, such as lack of transportation, lack of insurance, discrimination, stigma, fragmented healthcare systems, and lack of addiction treatment programs in local communities [4]. Furthermore, access to MOUD is racialized, with Black individuals being less likely to have access to buprenorphine in part due to neighborhood segregation [8].

Although social-distancing efforts during the COVID-19 pandemic allowed for greater flexibility in MOUD access –for example, allowing for more take-home methadone doses, and allowing removal of X-waiver prescription regulations for buprenorphine–unique limitations still remain for the provision of methadone therapy, reducing the availability of programs and settings where people can get treatment [9, 10]. As a result, low threshold interventions were developed to overcome such barriers and improve access to MOUD.

Mobile Addiction Treatment Units (MATU), sometimes known as mobile medication units, are defined as vans, vehicles, or otherwise portable clinics that provide low-threshold, low-barrier, community-based clinical care, and walk-in services for addiction treatment including MOUD and harm reduction practices to people experiencing barriers to office-based care. Low-threshold MATUs are an increasingly popular approach to addiction care for these underserved populations, acting as a point of entry for care [11]. The resources that these mobile units offer include safer consumption supplies, naloxone, fentanyl test strips, medication for substance use disorder treatment, and a wide range of primary and preventative care [12]. Typically, MATUs focus on providing access to traditionally hard-to-reach groups, such as people who inject drugs (PWIDs), persons experiencing homelessness, the uninsured [13], and racially minoritized individuals [12]. Mobile treatment offers the advantage of conducting outreach beyond the traditional office-based clinic into the communities where people with opioid use disorder (OUD) live and congregate [14]. Thus, MATUs are a strategy that may improve access to addiction treatment including MOUD, particularly among marginalized populations. However, there is no comprehensive scoping review focused on synthesizing and analyzing the research on implementation and outcomes of MATUs in the U.S.

Given this knowledge gap, we sought to conduct a narrative review of low-threshold MATUs to form a cohesive picture of the successes and challenges in this method of addiction care delivery. More specifically, our study’s primary objectives were threefold. First, we aimed to evaluate MATU program reach by assessing how effectively Mobile Addiction Treatment Units engaged and served their target populations, particularly individuals who are vulnerable, underserved, or experiencing homelessness. This included analyzing demographic and geographic data to determine whether the units were reaching communities with high levels of need and limited access to traditional healthcare. Second, we sought to evaluate MATU program effectiveness by examining the impact of the services on patient health outcomes, treatment initiation and retention, and overall engagement in care. Third, we evaluated MATU program implementation to understand the operational factors that influenced program delivery. This involved identifying strengths and challenges related to staffing, resource allocation, service delivery models, and external disruptions such as the COVID-19 pandemic, as well as assessing the adaptability, feasibility, and sustainability of MATUs as a mobile care model.

## Methods

This review is reported per the Preferred Reporting Items of Systematic Reviews and Meta-Analyses for Scoping Review (PRISMA-ScR) guidelines [15].

### Search strategy and eligibility criteria

We identified studies examining Mobile Addiction Treatment Units in the United States by searching electronic databases MEDLINE (Ovid), Embase (Elsevier), PsycINFO (EBSCO), and Web of Science Core Collection (Clarivate). The search was designed to retrieve studies including any mobile treatment modalities for opioid use disorder (OUD) and substance use disorders (SUDs) by combining terms for OUD and SUD treatments with mobile treatment modalities (Supplementary Material Appendix A). The search was conducted on April 4, 2024, without date restrictions. Controlled vocabulary terms were included when available.

#### Inclusion criteria

We used Covidence to manage and organize citations [16]. Two reviewers independently screened the titles and abstracts of the records retrieved in the search. Records were selected for full-text review if their abstract referenced any mobile modality for substance use treatments, including vans, treatment in patients’ residences, street-based counseling, street-based telehealth appointments, and therapies such as methadone, buprenorphine, and SUD counseling.

Of those selected for full-text analysis, we included quantitative and qualitative articles and brief reports that contained findings related to the intersection of substance use disorders and MATUs.

#### Exclusion criteria

Throughout the record screening process, we excluded review articles, opinion articles lacking primary data, theoretical articles, abstracts and dissertations, textbook chapters, studies conducted outside of the United States, and studies focused solely on other mobile harm-reduction interventions without offering treatment for SUD.

### Data extraction and synthesis

We developed a standardized template for data extraction. Two reviewers independently extracted data from selected articles, including study details (e.g., author, year of publication, setting), methods, inclusion/exclusion criteria, and findings and outcomes pertaining to the sustainability, implementation, and engagement of the MATUs. For sustainability, reviewers examined the resources, such as personnel and equipment, allocated for each intervention and the effects of external stressors such as the COVID-19 pandemic, and recorded the duration each intervention was studied; all relevant themes addressed by each study were subsequently in our summary of outcomes. To address implementation, reviewers noted any unique processes used, listed the barriers to implementation, and documented the target population demographics and region for each study. In terms of assessing engagement, reviewers tracked the number of individuals reached via each intervention across both treatment initiation and retention, the demographics of patients using these services, measures of physical and mental health, and reductions in substance use-related harms. Discrepancies in extraction were resolved by consensus after discussion with a third author.

**Fig. 1:**
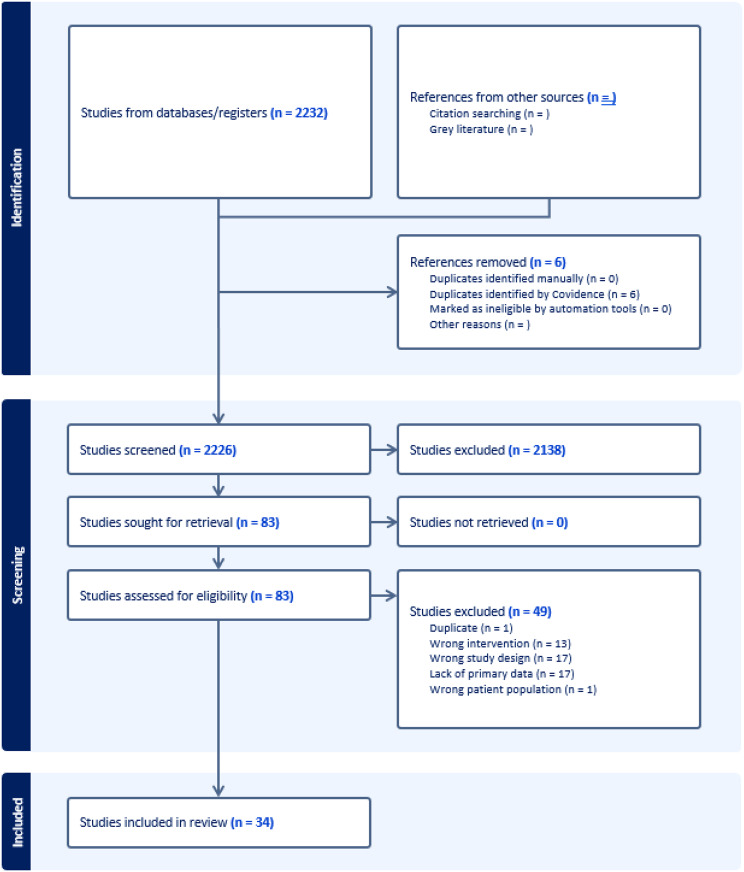
PRISMA diagram

We utilized a narrative synthesis approach to organize and present relevant findings for narrative and qualitative articles. This approach involves textual summaries and explanations of findings, which are synthesized through thematic analysis to explore relationships among studies and assess strength of evidence [17]. Emergent patterns were synthesized and narratively reported, and studies were thematically grouped by the setting and context of interventions and the type of sponsoring institution leading the program (Fig. 1).

## Results

### Research summary

Our literature review found 34 studies of MATUs, encompassing a range of interventions, target populations, and study designs. These studies varied in their methodologies, including randomized controlled trials (RCTs), observational studies, cohort studies, simulations, and mixed-methods research. To better characterize the sample for each study, key demographic and background variables including age, gender, race/ethnicity, geographic location, populations of focus (e.g., incarcerated individuals, adolescents, HIV-positive individuals), and other study-specific inclusion criteria were extracted. These relevant data are outlined in the “Population Description” column of Table 2. All studies were conducted in the United States: the most common study locations were Baltimore, MD (10 studies), Boston, MA (4 studies), Philadelphia, PA (4 studies), and New Haven, CT (3 studies). Three studies were conducted during the COVID-19 pandemic [18–20]. Five studies were conducted primarily in a population experiencing homelessness [21–25]. Two studies were conducted primarily in populations with criminal justice involvement [26, 27]. Four studies were conducted primarily in youth or young-adult populations [18, 19, 22]. Overall, we found studies to cluster by 4 population groups: rural settings, people experiencing homelessness, criminal justice, and youth.

Of the 34 studies reviewed, all but one [28] assessed patient and/or provider outcomes following implementation of a MATU. Seven of the studies assessed patient retention in MATUs [23, 25, 29–33]. Nine of the studies reported problems or challenges associated with MATUs [19–21, 30, 34–38]. The 34 analyses contained a total of 59,548 study participants.

### Synthesis of findings

#### MATUs in rural locations

Three studies focused on MATUs in rural locations [28, 38, 39]. A cross-sectional survey study of MATU use in rural Eastern Kentucky found that a majority of participants were willing to use a MATU for treatment (76.5%), particularly if they had been recently treated with medication for Opioid Use Disorder [38]. Individuals involved in the criminal justice system or with recent overdose histories were less likely to use a MATU [38]. A qualitative interview study of MATU use in the Confederated Tribes of the Grand Ronde Community of Oregon found that participants thought MATUs were more convenient than traditional brick-and-mortar clinics and helped to destigmatize opioid agonist therapy. Clinic patients noted that the MATU could be improved by increasing accessibility, particularly for the disabled or elderly [39]. A simulation study investigating the impact of introducing MATUs in rural Louisiana found that introducing 10 new MATUs would bring 20% of rural Louisiana Medicaid beneficiaries with at least one claim of diagnosed opioid dependence an average of 24 miles closer to treatment [40].

#### MATUs in populations experiencing homelessness

Three cohort studies focused specifically on the impact of MATUs in populations experiencing homelessness. A cohort study of a MATU based in Houston, Texas, found that the program offering counseling, medication stabilization, and hospitalization referral for a majority of patients (52% of females, 74.5% of males), resulted in female patients (33%) being more likely to become employed, and find stable housing (39%) than males (~10%) [22]. Similarly, a study of MATUs serving individuals experiencing homelessness in Philadelphia, PA found that retention rates in care were observed to be 61.2% at one month, 36.3% at three months, and 27.6% at five months [25].

Two qualitative studies investigated MATUs in populations experiencing homelessness in Massachusetts. Pepin et al. found that 29.4% of patients accessing the MATU were prescribed buprenorphine/naloxone, with a large portion of them receiving ongoing care [21]. Fine et al. found that the MATU attracted individuals primarily for specific services such as new needles (33.0%), food/drink (27.5%), and buprenorphine prescriptions (25.3%), though respondents ended up using a median of five services provided by the program [24].

One retrospective chart review assessed an innovative program in New Haven CT where community-based social workers connected veterans experiencing homelessness to buprenorphine prescribers via telehealth, including from homeless shelters and transitional housing sites, finding that retention rates in the program ranged from 100% at 1 month to 75% at 12 months, with an average treatment duration of 19.2 months [23].

#### MATUs in populations with criminal justice involvement

Two cohort studies assessed the use of MATUs in populations with criminal justice histories. One study of 487 formerly incarcerated individuals located in Oakland, CA found that participants reported decreased injection drug use, alcohol, crack, cocaine, and heroin use at both 6 and 12 months of follow-up. Participants also reported increased employment rates, from 13% at intake to 31% at 12 months. Outcomes were significantly improved among participants who engaged more frequently with MATU services [26]. Another study assessed a MATU program in Baltimore, MD focused primarily on initiating buprenorphine treatment for a demographic consisting mainly of African American males with previous criminal justice involvement. A majority of patients in the program (68%) completed multiple visits to the MATU, and 31.6% remained in treatment after 30 days [27].

#### Role of law enforcement in MATU reach and impact

Multiple studies addressed the complex role of law enforcement in influencing the implementation and effectiveness of MATU programs. Davis et al. examined barriers to care to four mobile clinics in Massachusetts through qualitative interviews, highlighting that police presence often negatively impacted MATU operations through disrupting encampments, contributing to patient mistrust, and objecting to services provided by clinics [41]. However, the authors also observed that the nature of police-provider relationships varied considerably by location, with some clinics reporting collaborative and supportive interactions [41]. Pepin et al. further noted that encampment clearances mediated by local law enforcement posed significant barriers to patient retention and continuity of care, including loss of medications and harm reduction materials [21].

#### MATUs in youth populations

One cohort study examined MATU use in a population of youth aged 15–25 experiencing homelessness, finding that the program effectively improved self-worth and mood stabilization and was successful in screening and treating STIs, as well as viral infections like hepatitis, HIV, and HPV [22]. Two studies examined MATU use in youth populations during the COVID-19 pandemic. A review assessed five different MATU programs for adolescents and youth with substance use disorders during the COVID-19 pandemic [19]. Across the five programs assessed, two reported no decrease in the number of visits recorded by the program during the pandemic. Three programs transitioned to telehealth, which increased patient access to care, but posed challenges for patients with serious mental illness who required in-person assessments such as regular urine testing. One case report examining a Baltimore, MD-based MATU serving young adults with OUD found that mobile van delivery of XR-MOUD during the pandemic offered advantages such as enhancing patient security and confidentiality [18]. However, authors reported that the lack of in-home settings prohibited participants’ family members from providing support for treatment adherence [18].

### Impact of COVID-19

The COVID-19 pandemic has had a profound impact on the operation and accessibility of MATUs. Three studies in our review were conducted during the pandemic, highlighting both challenges and opportunities [18, 20, 42]. The pandemic prompted regulatory changes, such as the expansion of telehealth services for SUD treatment, provision of take home medications, and waiver of prescription licensure requirements for medical providers [9, 10]. These changes have increased the feasibility and accessibility of MATUs. However, the pandemic also introduced operational challenges, including staffing shortages and supply chain disruptions, which affected service delivery [20, 42].

### Pharmacies as partners

One cohort study involved pharmacies as partners for implementation of a mobile buprenorphine treatment initiation program based near Baltimore City Jail [25]. In this study, the mobile van partnered with a nearby pharmacy to allow uninsured patients to receive their medication free of charge, with an identification system intended for vulnerable populations who may not have access to government-issued identification methods that are often required by states. As a result, patients were provided access to medication on the very day that they were ready to begin treatment, minimizing the delay between the stages of preparation and action [29]. Over the course of a year, the mobile van provided 190 with buprenorphine prescriptions, with nearly one-fifth of the participants noted to be uninsured and the vast majority of the participants with a history in the criminal justice system and unstably housed [25]. This novel partnership effectively facilitated the role of mobile vans in reaching the most underserved patients with minimal access to the healthcare system.

### Retention rates comparison

MATU retention rates suggest superior performance compared to traditional office-based settings when serving highly vulnerable populations, such as recently incarcerated or homeless populations, though they remain below general population benchmarks. For the general population receiving MOUD in conventional clinic settings, systematic reviews indicate retention rates of approximately 40–60% at six months, with community-based studies reporting 49% retention for buprenorphine and 58% for methadone at six months [43, 44]. Randomized controlled trials evaluating buprenorphine maintenance have documented retention figures between 39 and 59% over 6 months [44]. Unfortunately, no studies have measured retention rates in office-based addiction treatment programs for homeless or severely underserved populations at a national scale in the US. However, retention among vulnerable populations in traditional settings is substantially lower, with a pan-Canadian trial of highly vulnerable participants (50% experiencing homelessness) showing only 17.7% retention on buprenorphine/naloxone and 18.4% on methadone at 24 weeks [45]. In a local cohort study from Boston, Massachusetts, an office-based addiction treatment program for unstably housed patients reported a 45.1% one-month retention rate, and 21.7% retention rate at 6 months, comparable to the national Canadian study [46].

MATU programs demonstrated markedly higher engagement among similar vulnerable populations, with 30-day retention averaging 74.9% across seven studies (range: 31.6–100%), including strong performance among homeless populations (80.6%) and justice-involved individuals (62.7%) [23, 25, 27, 33] (See Table 1). While retention declined over time, with three-month rates averaging 31.3% [25, 32], several programs maintained substantial longer-term engagement, including 80.7% at one year for street medicine patients and 75% at 12 months among homeless veterans [23, 31]. These findings are particularly significant given that quantitative and qualitative evidence indicates MATU participants were predominantly disconnected from traditional healthcare, highly stigmatized, and had experienced barriers including transportation difficulties, insurance issues, and discrimination in conventional settings [37, 47]. The superior retention rates suggest that MATUs represent a critical intervention for populations who would otherwise remain untreated, providing accessible, low-threshold care that overcomes structural barriers inherent in traditional treatment modalities [36, 48].

**Table 1:** Summary of retention rates from scoping review

Article	30-Day retention rate and population description
Iheanacho (2020) [23]	100% (Veterans experiencing homelessness)
Krawczyk (2019) [27]	31.6% (Recently incarcerated individuals)
Langabeer (2020) [30]	88% (ED patients with OUD)
Martinez (2024) [37]	94% (PCARE Van program patients)
O’Gurek (2021) [25]	61.2% (Individuals experiencing homelessness)
Rosecrans (2022) [32]	56% (People who inject drugs)
Selitsky (2022) [33]	93.8% (Formerly incarcerated individuals)

Article	90-Day retention rate and population description
Kuo (2003) [49]	84% (Needle exchange program patients)
Langabeer (2020) [30]	56% (ED patients with OUD)
O’Gurek (2021) [25]	36.6.2% (Individuals experiencing homelessness)
Selitsky (2022) [33]	80% (Formerly incarcerated individuals)

## Discussion

Overall, our review of 34 articles found that MATUs had positive effects on participants. The studies, primarily observational with small sample sizes across various settings, showed that MATUs were associated with reduced substance use, crime, and stress, as well as improved self-rated health and employment. Mobile units provided medications for opioid use disorder, psychiatric interventions, harm reduction services, and STI screenings, particularly benefiting homeless populations and those with limited access to healthcare [14, 21–25, 47, 48, 50, 51]. Programs demonstrated high engagement and retention rates, especially when employing respectful and supportive staff, comprehensive care models, and community-based services [23, 25, 29–33]. MATUs effectively reached vulnerable and underserved groups, offering holistic care that integrated harm reduction, primary care, and mental health services. These units showed promise in improving health outcomes, reducing stigma, and addressing social determinants of health.

Despite these successes, the literature on MATUs described several challenges that they face. Issues such as maintaining patient engagement, managing logistical and regulatory barriers, and ensuring financial sustainability were common [34]. Limited space, staffing, and resources hindered the delivery of consistent and comprehensive care [37]. The transient nature of homelessness and external disruptions like encampment removals impacted retention rates and continuity of care [25]. Additionally, the COVID-19 pandemic exacerbated difficulties in service delivery, although telehealth adaptations helped maintain treatment access [19, 52].

While innovative and promising in their approach to addressing SUDs in underserved populations, we found that MATUs have been primarily studied through qualitative methods like surveys and interviews; 19 out of the 34 selected articles focused on pilot programs or relied heavily on narrative and qualitative data obtained from interviews of a limited subset of care providers and participants. While these studies provide valuable insights into the subjective experiences and perceived benefits of MATUs, they often lack the robust data necessary to comprehensively assess their long-term impact. Future research must prioritize large-scale, longitudinal studies that employ rigorous quantitative and qualitative methodologies to evaluate the effectiveness of MATUs, while accounting for lower retention rates in participants.

### Outreach and stigma reduction

A recurring theme in the reviewed studies was the reduction of stigma experienced by patients utilizing MATUs compared to traditional office-based settings, consistent with prior qualitative findings [53]. Many patients reported feeling more comfortable and less judged when receiving treatment in the more informal and accessible environments provided by MATUs. For instance, participants from the Confederated Tribes of the Grand Ronde Community of Oregon highlighted the convenience and destigmatizing nature of MATUs, sensitizing community members to the services they provide and the patients served [39]. Two studies even highlighted the adoption of their program as a preferred point of care for the patients they served, despite having access to brick and mortar clinics [20, 37]. The mission of MATUs to deliver treatment to underserved community members aligns with the need to address stigma, which often deters individuals from seeking care [54, 55]. This aspect of MATUs can be leveraged in future program designs to maximize patient engagement and retention.

As noted above, access to MOUD is racialized, with neighborhood segregation shaping who has access to buprenorphine versus methadone in brick-and-mortar settings [8]. MATUs may hold promise for combating structural racism by allowing MOUD and other addiction services to be brought to racialized minority groups at risk for overdose death [12].

Furthermore, one study observed a decrease in overall crime rates in areas served by MATUs compared to control groups, suggesting that these units may contribute positively to community safety [56]. This result could potentially mitigate concerns about increased criminal activity and further reduce stigma surrounding SUDs and MATUs. However, understanding and addressing community barriers remains crucial for the successful implementation and acceptance of MATUs. Future studies should explore community perceptions in greater depth to devise strategies that foster better integration and reduce any potential backlash.

### Financial sustainability and implementation challenges

Several studies highlighted challenges regarding the financial sustainability of MATUs [34, 35, 37]. One of the primary obstacles was the lack of a funding infrastructure that accommodates the unique service delivery model of MATUs. Public health insurance programs such as Medicare and Medicaid have yet to fully adapt their billing services to support MATUs, creating barriers for healthcare providers [57]. Addressing these billing challenges and integrating MATU operations into a sustainable funding model appear key to ensuring these programs’ long-term viability. Further studies assessing policy changes and funding mechanisms tailored to mobile delivery systems of MOUD should be developed to support the integration of MATUs into the broader healthcare system.

Innovative implementation modalities for MATUs, like the Community Health Care Van in New Haven, coupled MOUD with primary care for the broader population, suggesting a viable strategy to enhance community acceptance and support while expanding overall healthcare services in underserved communities [58]. This approach not only facilitates flexible and sustainable modes of service delivery but also helps in reaching underserved populations, while offering health services available to the broader community, increasing their likelihood of acceptance by the broader community.

### Methadone services in MATUs: an untapped opportunity

While the majority of studies in this review focused on buprenorphine delivery through MATUs, methadone services represented a smaller but significant portion of the interventions. Of the 34 studies reviewed, only five programs offered methadone services [14, 20, 35, 39, 49]. Additionally, Gibbons et al. conducted a simulation study specifically examining the potential impact of mobile methadone units in rural Louisiana, finding that 10 new mobile units could bring 20% of rural Medicaid beneficiaries an average of 24 miles closer to treatment [40].

However, mobile methadone services represent a largely untapped opportunity to address one of the most significant barriers in addiction treatment. Methadone remains the only medication for opioid use disorder that cannot be prescribed in office-based settings and requires daily visits to specialized Opioid Treatment Programs (OTPs), disrupting daily activities of patients such as employment and family responsibilities [59]. Recent evidence demonstrates that geographic barriers affect 18.2% of the US population who lack access to methadone clinics [60], and that transportation issues are the second most common reason for treatment dropout [61], mobile methadone services could facilitate retention and induction of underserved and socially vulnerable populations while allowing them to maintain quality of life and normal daily activities.

### General interpretation and implications for future research

The findings of this scoping review underscore the potential of MATUs to enhance access to addiction treatment for underserved populations and to improve retention of MAT in populations disconnected from traditional health services. However, the current evidence base is limited by the reliance on pilot studies and qualitative data. Future research should focus on conducting large-scale, quantitative and qualitative studies to assess the long-term impact of MATUs. Additionally, the integration of MATUs with primary care services presents a promising avenue for expanding their reach and sustainability [58]. Further studies should also be conducted in diverse geographical locations, particularly in rural areas, to better understand and address the unique challenges and benefits of MATUs in these settings.

Policy and regulatory changes, such as the removal of the X-waiver and the expansion of telehealth services, have created new opportunities for MATUs. Research initiatives like the NIH HEAL Initiative encourage the exploration of innovative SUD treatment delivery modalities, providing a platform for further study and demonstration of MATUs [62]. By addressing the identified gaps and limitations, future research can provide stronger evidence to support the implementation and legitimization of MATUs as a vital component of addiction treatment in the United States.

## Conclusion

This narrative review comprehensively examined Mobile Addiction Treatment Units (MATUs) as an innovative approach to expanding access to addiction treatment for underserved populations in the United States. Through analysis of 34 studies encompassing diverse methodologies and settings, we addressed our three primary objectives: evaluating MATU program reach, effectiveness, and implementation. Our findings show that MATUs successfully reached vulnerable populations who face significant barriers to traditional care, including individuals experiencing homelessness (featured in seven studies) [21–25, 47, 48], those with criminal justice involvement (three studies) [26, 27, 33], youth populations (two studies) [18, 22], and rural communities (three studies) [38–40]. The geographic concentration of studies in urban centers (See Table 2) highlights both the proven feasibility of these programs in high-need areas and the critical gap in rural implementation that warrants further investigation.

**Table 2:** Studies included in review

Author, Year, and Location	Study Design	Sample Size	Population description	Addiction treatment offered	Retention Rates	Relevant Outcomes
Bartholomew 2022 [63] Florida, Miami-Dade County	Semi-structured qualitative interviews and quantitative demographic assessment.	30 participants	Black patients who inject drugs	Buprenorphine administration and initiation	Retention rates not reported	Participants found the mobile unit more accessible than traditional treatment programs. They believed it effectively addressed transportation, cost, and stigma barriers. Participants emphasized the importance of nonjudgmental and approachable staff with lived drug use experience.
Bowser 2010 [26] Oakland, California	Cohort study	487 participants	Men and women on probation who have a history of substance use	Intensive case management, counseling, and substance abuse education.	Retention rates not reported	Clients reported reduction in alcohol, crack, cocaine, and heroin use, and a decrease in the number of crimes committed. Despite an increase in days spent in jail, injection drug use declined. Participants experienced reduced stress from drug use and improved self-rated health. Employment rates also increased from 13% at intake to 31% at 12 months.
Busen 2008 [22] Houston, Texas	Cohort study	95 participants	Youth aged 15–25 experiencing homelessness utilizing a mobile medical unit	Provided psychotropic medications for psychiatric stabilization and intensive psychotherapy and counseling services.	Used for an average 14 months. No retention rates reported	The participants had multiple psychiatric disturbances (76%) and high rates of substance use (46.8%) linked to depression, molestation, and abandonment. The program improved self-worth and mood stabilization.
Chatterjee 2024 [34] Boston, MA and Ohio	Qualitative Interviews	5 mobile units, 11 staff members interviewed	Program staff from mobile MOUD clinics	Buprenorphine administration and methadone referral. The mobile units also provided telemedicine access for MOUD follow-up.	Retention rates not reported	Community engagement and partnerships were crucial for the success of mobile treatment services. Significant challenges included stigma, limited resources, emergency repairs, regulatory challenges and financial sustainability. Participants noted that funding from the HEALing Communities Study facilitated the establishment of mobile units. Mobile treatment units were seen as innovative solutions that expanded access to medications, promoted equity in treatment, and reduced stigma [41].
Davis 2024 [41] Massachusetts	Qualitative Interviews	4 participants	Clinical providers at four mobile addiction clinics in Massachusetts	Buprenorphine induction	Retention rates not reported	The patient population was largely unhoused and disconnected from traditional healthcare. Major challenges included finding a location to park, relations with police and poor continuity due to a model built on unscheduled visits.
Fine 2021 [24] Massachusetts	Qualitative Interviews	119	Patients experiencing homelessness receiving care from a mobile addiction-focused outreach program.	Buprenorphine	Not reported, but 95.6% of participants said they would return for care.	25.3% of participants received buprenorphine prescriptions, amongst other health services offered. 98.9% of respondents felt respected by the program staff. Additionally, 70% reported to decrease their drug or alcohol use because of the program, and would recommend it to their friends. The mobile program was preferred over office-based care, due to improved waiting times, and focus on addiction treatment and homelessness. Prior to the program, 20.9% had not seen a provider for over three years and 62% reported past unfair treatment due to their housing status, substance use, or inability to pay.
Fine [47 2023] Boston, MA	Non-randomised quasi-experimental study	138 participants	Adults who engaged with the mobile addiction program run by Boston Health Care for the Homeless	Buprenorphine induction and maintenance	Mean number of 3.1 encounters. Retention rates not reported.	Inpatient hospitalizations for the mobile program cohort increased significantly from 2.2 to 3.0 (*p* = 0.04), but remained stable at 2.5 in the control cohort (*p* = 0.82). Overall, the mobile addiction program did not significantly affect healthcare utilization compared to a large Health Care for the Homeless program in Boston, MA.
Fixler 2024 [56] PA, Pittsburgh	Non-randomised experimental study	84 census blocks	Pittsburgh residents in neighborhoods with high overdose data, crime statistics, new HepC and HIV diagnoses, absence of community resources and treatment programs.	Buprenorphine administration and initiation	Retention rates not reported	The study found a significant decrease in quarterly arrests after treatment was introduced, with a 37.7% reduction in block groups within 1 mile of a clinic and an 18% reduction in more distant areas. After the clinics were established, total arrests dropped by 34.13% and non-drug-related arrests by 22.29% within one mile of the clinics compared to areas further away. There was no significant increase in crime near the clinics, countering concerns that harm reduction services might attract local crime.
Gibbons 2024 [40] Louisiana	Simulation	43,341 participants	Beneficiaries of Louisiana’s Medicaid program with at least one claim of diagnosed opioid dependence.	Methadone provision	Retention rates not reported	The study predicted that introducing 10 new mobile methadone units in Louisiana would increase the net MOUD (Medication for Opioid Use Disorder) treatment rate by 0.54–2.39% points. Exclusively serving rural areas, these units could boost rural MOUD treatment by 8.54–13.67% points, bringing roughly 20% of rural beneficiaries an average of 24 miles closer to treatment. Mobile methadone units are a promising innovation for increasing methadone use, especially in underserved rural locations.
Gibson 2017 [58] New Haven, CT	Cohort study	8404 participants	Patients from medically marginalized communities served by The New Haven Community Health Care Van.	Buprenorphine maintenance or methadone referral	Retention rates not reported.	Mental health services accounted for 11.4% of total visits, primarily provided as ancillary counseling for patients on buprenorphine maintenance therapy. Higher healthcare utilization was correlated to patients being foreign-born, injecting drugs, having hypertension, and being HIV+. Fewer visits were associated with not completing high school and engaging in sex work. The program utilized Spanish interpreter services.
Gibson 2014 [57] New Haven, CT	Cross sectional study	8404 participants	Patients of the community healthcare van (CHCV).	Buprenorphine maintenance therapy.	45.2% of patients receiving buprenorphine treatment made 11 visits or more in the span of 8 years.	Mobile clinics attract clients despite being geographically close to other addiction treatment facilities. Indeed, participants traveling over fifty miles were significantly more likely to be homeless (40.3%, *p* = 0.03) and socially marginalized. 94.4% of high-frequency patients receiving MAT were foreign-born, 50% were unstably housed, 85% unemployed, and 75% had an HIV+ status. Authors expressed challenges from lack of provisions for mobile clinics by the ACA.
Grieb 2022 [64] Baltimore, MD	Qualitative interviews	31 interviewees: 16 mobile health clinic clients and 15 syringe service users who are not clients	PWUD accessing mobile health clinic partnered with syringe services program.	Buprenorphine-based MOUD, overdose prevention and response training, and case management	Retention rates not reported	People who use drugs viewed a mobile health clinic in their neighborhood positively, seeing benefits such as improved access to healthcare services, low-threshold buprenorphine dispensation, and services without drug-use stigma. Word-of-mouth was the primary way they learned about the clinic, which limited their access due to incomplete information about the services provided. Non-clients typically used methadone instead of buprenorphine.
Hall 2014 [14] New Jersey	Cross sectional study	2,259 participants	Individuals enrolled in NJ-MATI program	Methadone maintenance, buprenorphine induction and maintenance, and referral to office-based care.	Retention rates not reported	NJ-MATI (New Jersey Medication-Assisted Treatment Initiative) clients, were more likely to be African American, homeless, and uninsured compared to traditional methadone clients. Public funding eliminated affordability as a barrier for treatment participation. NJ-MATI clients had higher rates of co-occurring mental illness (OR = 1.6, 95% CI = 1.3, 2.1) compared to traditional methadone clients but showed no significant difference in court supervision. Compared to non-MAT clients, NJ-MATI clients were more likely to have used drugs prior to age 18 (OR = 1.3, 95% CI = 1.1, 1.6), to report IVDU (OR = 6.5, 95% CI = 5.4, 7.6), to report daily drug use (OR = 11.5, 95% CI = 9.1, 14.5), and less likely to report past month treatment (OR = 0.4, 95% CI = 0.2, 0.5) or prior AA/NA attendance (OR = 0.5, 95% CI 0.4, 0.6).
Harris 2022 [31] Baltimore, MD	Cross sectional study	150 participants	Patients of The Spot, a mobile street medicine program in Baltimore.	Telemedicine buprenorphine treatment services	80.7% (*n* = 121) of patients remained engaged in treatment at one year	Demographic analysis showed no significant differences in age, gender, race, or ethnicity between patients who transitioned and those lost to follow-up. Telemedicine engagement was supported by flexible service delivery and extended prescription lengths, contributing to high retention rates comparable to in-person services.
Hoffman 2024 [39] Oregon	Qualitative Interviews	17 participants: 11 patients, 5 staff, 1 state opioid treatment authority	The Confederated Tribes of the Grand Ronde Community of Oregon	Methadone or buprenorphine maintenance and induction	Retention rates not reported	Participants acknowledged the Mobile Medical Unit (MMU) for making treatment more accessible. Less stigmatized and convenient. A significant barrier for the staff was needing a physician present for all intakes. Some patients suggested improved accessibility,for the disabled or elderly. Overall, the Great Circle MMU enhanced access to opioid agonist therapy for both American Indians/Alaska Natives and non-natives in rural communities.
Iheanacho 2020 [23] New Haven, CT	Retrospective chart review	36 participants	Veterans experiencing homelessness	Mobile technology and FaceTime in addition to existing community-based case management programs to provide buprenorphine treatment	Mean retention in treatment was 19.2 months (SD = 10.2) in M-CAT and 36 months (SD = 27.6) in BUP clinic. At the endpoint, 66.7% (*n* = 8) in M-CAT and 100% (*n* = 24) in BUP clinic remained on BUP.	The team uses a mobile video conferencing app to facilitate psychiatric evaluations and initiate buprenorphine (BUP) treatment, addressing barriers such as transportation and missed appointments associated with clinic-based care. Retention rates in the M-CAT program ranged from 100% at 1 month to 75% at 12 months, with an average treatment duration of 19.2 months. While retention rates were lower compared to the traditional BUP clinic (100% retention at endpoint), they were higher than similar low-barrier interventions for veterans and non-veterans with OUD who are homeless.
Krawczyk 2019 [27] Baltimore, MD.	Cohort study	190 participants	Patients with OUD who were recently incarcerated and the general public.	Same-day visit buprenorphine initiation and maintenance from a van. The program covered MAT prescription copays.	31.6% retention 30 days after initiation.	94.5% of the study’s population had been incarcerated. 85% were African American males with a mean age of 44.1 years and average opioid use of 24.0 years. 70.8% had unstable housing, and 15.6% were uninsured. 32.1% had a history of overdose.
Kuo 2003 [49] Baltimore, Maryland	Non-randomised experimental study	114 participants	Patients of a Needle exchange program (NEP) in Baltimore, MD.	Medical addiction treatment using levomethadyl acetate hydrochloride (LAAM)	84% retention in drug treatment program over 90 days.	Participants in the program showed significant reduction in drug, alcohol, and legal issues as measured by ASI scores after one month compared to non-participants, (*p* < 0.0001),(*p* = 0.003), (*p* = 0.004) respectively. Specifically, there was a 31% decrease in opiate-positive urine tests and a 22% decrease in cocaine-positive tests between the first and third months of treatment.
Langabeer 2020 [30] Houston, TX	Cross sectional study	103 participants	Patients at the ED of Memorial Hermann Hospital (MHH) with OUD and lack of current enrollment in OUD treatment.	Buprenorphine management, addiction counseling, and social assistance in participant’s home	88% retention at 30 days, 56% retention at 90 days	33% of contacted individuals enrolled in the treatment program.
Lowenstein 2023 [36] Philadelphia, PA	Qualitative Interviews	36 participants	Patients of low-barrier buprenorphine program	Buprenorphine induction and maintenance and referral to outpatient medical programs.	Retention rates not reported	Participants highlighted positive characteristics of the program, including convenience, flexibility, and the supportive, empathetic care team, which contrasted with participants’ past treatment experiences. Barriers included long wait times, inclement weather, lack of privacy, and limited ability for patients to access mental health support services.
Martinez 2024 [37] Baltimore, MD	Qualitative Interviews	26 participants	Patients and staff members of the PCARE Van program.	Buprenorphine treatment, assistance with transition to clinic-based care	Thirty-day program retention is 94%.	Patients preferred long-term care at low-threshold programs, citing positive relationships, predictability, and sense of community. However, staff highlighted challenges in maintaining this model due to limited van staffing, space, and few clinic-based partners to meet patient needs. Key themes included the importance of respectful, low-threshold care, patient preference for continuity, harms of rigid care models, and the inadequacies of the system versus individual shortcomings.
Messmer 2023 [65] Chicago, IL	Cohort study	587 participants	Patients in traditionally medically un­derserved Chicago neighborhoods with the highest overdose rates	Buprenorphine treatment, referral to outpatient medical programs.	Retention rates not reported	Multiple patients initially presented to the mobile unit for medical issues unrelated to OUD (most commonly COVID-19 vaccination, 42.4%) and then ultimately returned to initiate MOUD. Buprenorphine treatment was a reason for 51.6% of follow-up visits, with some patients preferring to continue care at the mobile unit rather than traditional healthcare facilities.
O’Gurek 2021 [25] Philadelphia, PA	Cohort study	147 participants	Individuals predominantly experiencing homelessness (90%) and opioid use disorder	Buprenorphine treatment, education and counseling services	Retention rates were 61.2% at 1 month, 36.6% at 3 months, and 27.6% at 5 months.	The program successfully engaged a population experiencing concurrent homelessness (90%), intravenous opioid use (67%), and prior treatment for substance use disorder (92%). Lapses in care were common but short (average time of lapse in care across patients = 1.5 weeks). Barriers such as the transient nature of homelessness likely contributed to lower retention rates.
p. 2024 [42] Baltimore, MD	Randomized controlled trial. Comparison between expanded MOUD services and needle exchange vs solely needle exchange and naloxone distribution	720 participants	People in 12 Baltimore sites with high drug use activity and served by the city’s mobile needle exchange program	Buprenorphine initiation and maintenance	Retention rates not reported	Over a median of 10.4 months, the ICV buprenorphine/naloxone initiation for 540 clients (74%). The trial found no evidence that weekly neighborhood visits from a mobile health van providing injection-drug-focused services improved access to services and outcomes among people who injected drugs, compared to usual services.
Peddireddy 2024 [38] Lexington, Kentucky	Cross sectional study	174 participants	Individuals in rural Eastern Kentucky	Potential offering of buprenorphine and naltrexone.	Study measures interest in potential MATU programs. Therefore, no retention rates are reported.	76.5% of participants were willing to use a Mobile Treatment Unit (MTU). Those recently treated with Medication for Opioid Use Disorder (MOUD) were six times more likely to be willing, while willingness was 81% lower among those who had overdosed in the past six months.
Pepin 2023 [21] Worcester, MA	Qualitative Interviews	330 participants	Patients with opioid use disorders who are experiencing homelessness	Buprenorphine/naloxone prescription and induction	69.4% made more than 1 visit. 20% made more than 10 visits 6.7% made more than 20 visits.	In two years, the team had 4567 encounters. One of the major challenges highlighted was managing patient care disruptions caused by forced removals of encampments by city authorities, which resulted in the loss of patient belongings and medications.
Regis 2020 [48] Boston, MA	Mixed methods.	119 participants	Vulnerable populations including individuals experiencing homelessness at risk of overdose.	Buprenorphine induction, naltrexone administration, and methadone referral.	Retention rates not reported. However, authors report increased demand for services throughout the pilot.	In its initial 10 months, the program engaged > 3,800 individuals who use drugs, resulting in 308 clinical encounters. Qualitative interviews indicated a positive reception by stigmatized individuals in traditional healthcare settings. Participants highlighted convenience and approachability as a major benefit of the model.
Rosecrans 2022 [32] Baltimore, MD	Cohort study	569 participants	People who inject drugs, at risk of drug overdose and infectious diseases.	Buprenorphine induction and maintenance integrated with health care services.	Buprenorphine treatment retention was 56.0% at one month and 26.2% at three months.	73.9% of participants received buprenorphine. Buprenorphine retention rates were higher in black patients and those with hepatitis C. Patients on buprenorphine were more likely to be tested and treated for HIV, hepatitis C, and sexually transmitted infections.
Rosenblum 2002 [50] New York, NY	Non-randomised experimental study	250 participants	Patients who had at least one encounter with the mobile medical van in 1997.	Intensive case management with food and transportation assistance as well as medical referrals to addiction treatment.	80% followed up after first visit	The experimental group showed fewer days of “crack” use (4.1 vs. 2.2 days, *p* < 0.05), fewer days homeless (13.2 vs. 10.2 days), and fewer health complaints (6.3 vs. 4.8) over 30 days compared to controls.
Selitsky 2022 [33] Baltimore, Maryland	Cohort study	566 patients	Formerly incarcerated individuals who use opiates	Buprenorphine administration and prescription	93.8% retention for 30 days and 80% retention for 90 days	Higher daily doses of sublingual buprenorphine ( > 16 mg) were significantly associated with increased odds of patients remaining engaged in treatment for at least 30 days (*p* < 0.001). Conversely, female patients had lower odds of 30-day retention (AOR = 0.43, 95% CI = 0.19–0.95, *p* = 0.04), as did those experiencing homelessness (AOR = 0.23, 95% CI = 0.19–0.52, *p* < 0.001), and those with prior buprenorphine treatment history.
Stewart 2023 [35] Philadelphia, PA	Qualitative interviews	7 participants	Leadership and staff of mobile OUD care units.	Buprenorphine prescriptions, naltrexone administration, methadone referral.	Retention rates not reported	Mobile Opioid Care Units (MOCUs) operated 7 to 35 hours per week and served 20 to over 100 individuals with varying touchpoints per patient. Practical challenges included limited space, patient accessibility, and volume constraints. Funding is a significant barrier, with most units relying on grants and inadequate medical service reimbursements.
Stewart 2021 [66] Philadelphia, PA	Cohort study	468 participants	Individuals with OUD in neighborhoods with a high opioid overdose mortality.	Free and immediate transportation to physical addiction treatment site, where methadone is dispensed.	Retention rates not reported.	The transportation group had a lower probability, before being engaged, of using treatment services than the walk-in control group. The study reported a 32% increase in methadone adherence over 3 months in the experimental group compared to the walk-in non transportation group.
Suen 2023 [20] San Francisco, CA	Qualitative Interviews	30 participants	Providers and patients of mobile addiction treatment programs in SF	Methadone prescription and counseling	Retention rates not reported.	The methadone van service faced logistical challenges with medication storage and Wi-Fi connectivity for electronic health records (EHR). Patients appreciated the efficiency, accessibility, and positive environment from the van staff. Counselors encountered difficulties with telehealth counseling, especially for patients without phones, and maintaining contact with patients.
Wenzel 2021 [18] Baltimore, Maryland	Case report	Not reported	Young adults with opioid use disorder (OUD)	Brief psychotherapy and administration of long acting buprenorphine or naltrexone	Retention rates not reported.	Mobile van delivery of MOUD during the COVID-19 pandemic, reduced COVID-19 exposure risk while preserving the confidentiality of participants. However, one limitation was reduced engagement from participants’ relatives, who remained at home.

Regarding effectiveness, MATUs demonstrated promising outcomes across multiple domains. Programs achieved retention rates ranging from 31.6% to 100% at 30 days, with some innovative approaches like telemedicine-supported street medicine achieving 80.7% retention at one year [31]. Beyond medication provision, MATUs effectively integrated comprehensive services including harm reduction, primary care, mental health support, and social services, addressing the complex needs of marginalized populations [22, 31, 49, 57, 58, 64]. The implementation analysis revealed both significant strengths and persistent challenges. While MATUs reduced stigma, improved accessibility, and fostered patient engagement through low-threshold, community-based approaches, they faced substantial barriers including financial sustainability, regulatory constraints, staffing limitations, and logistical challenges exacerbated by external factors like encampment clearances and the COVID-19 pandemic [18, 21, 34, 35, 41, 57].

Despite these demonstrated successes, our review identified critical limitations in the existing evidence base that must be addressed to advance the field. The predominance of single-site program evaluations, small qualitative studies, limited sample sizes, and short-term follow-up periods constrains our ability to draw definitive conclusions about long-term effectiveness and scalability. Future research must prioritize large-scale, multi-site randomized controlled trials with extended follow-up periods to establish robust evidence for MATU effectiveness. Additionally, the untapped potential of mobile methadone services, which showed dramatic improvements in retention rates when implemented, deserves particular attention given the unique regulatory barriers that make methadone the most difficult MOUD to access [59, 67]. As policymakers and healthcare systems seek innovative solutions to the ongoing overdose crisis, MATUs represent a promising intervention that bridges the gap between traditional clinic-based care and the lived realities of people who use drugs. However, realizing this potential will require sustained commitment to addressing implementation challenges, securing stable funding mechanisms, and conducting rigorous research that can guide evidence-based expansion of these vital services to all communities in need.

## Electronic supplementary material

Below is the link to the electronic supplementary material.

Supplementary Material 1

## Data Availability

No datasets were generated or analysed during the current study.
