# Supplementary material for: Mobile addiction treatment units: a narrative review
Source: Addict Sci Clin Pract. 2025 Dec 24;20:99. doi: 10.1186/s13722-025-00619-1 (PMC12729169; doi:10.1186/s13722-025-00619-1)
Supplement: Supplementary file 1 — Supplementary Material 1 [file 13722_2025_619_MOESM1_ESM.docx]

| Author(s) (Year)  Location | Study Design | Population description | Relevant Outcomes |
| --- | --- | --- | --- |
| Peddireddy, 2024[^40^](https://www.zotero.org/google-docs/?TaJJ4m) Lexington, Kentucky | Cross sectional study | Individuals in rural Eastern Kentucky, at the epicenter of the  US rural opioid epidemic | 76.5% of participants were willing to use a Mobile Treatment Unit (MTU). Those recently treated with Medication for Opioid Use Disorder (MOUD) were six times more likely to be willing, while willingness was 73% lower among those under community supervision and 81% lower among those who had overdosed in the past six months. The study shows MTUs are generally acceptable in rural US areas but face challenges among those involved in the criminal justice system or with recent overdose histories. |
| Page[^52^](https://www.zotero.org/google-docs/?HkC6jB) 2024 Baltimore, MD | Randomized controlled trial | People in 12 neighborhood sites in Baltimore with high drug use activity and served by the city's mobile NEP | Over a median of 10.4 months, the ICV provided services to 734 unique clients across six intervention sites. This included HIV/hepatitis C testing for 577 clients (78.6%) and buprenorphine/naloxone initiation for 540 clients (74%). The composite score decreased at 7 months, but there was no significant difference. A cluster-randomized trial in Baltimore, MD, found no evidence that weekly neighborhood visits from a mobile health van providing injection-drug-focused services improved access to services and outcomes among people who injected drugs, compared to usual services. |
| Martinez[^39^](https://www.zotero.org/google-docs/?6aok34) 2024 Baltimore, MD | Qualitative research/Interviews | Patients and staff members of the PCARE Van program in Baltimore, MD. | Several patient participants expressed a desire to remain at the van for continued connection and predictability, valuing the positive relationships and sense of community. Patients preferred long-term care at low-threshold programs. However, staff highlighted challenges in maintaining this model due to limited van staffing, space, and few clinic-based partners to meet patient needs. System-level barriers and limited resources further complicated transitions. Key themes included the importance of respectful, low-threshold care, patient preference for continuity, harms of rigid care models, and the inadequacies of the system versus individual shortcomings. |
| Hoffman[^41^](https://www.zotero.org/google-docs/?exmt4M) 2024 Oregon | Qualitative research/Interviews | The Confederated Tribes of the Grand Ronde Community of Oregon | Participants acknowledged the Mobile Medical Unit (MMU) for making treatment more accessible and convenient. They noted that the MMU's mobility and community presence reduced the stigma associated with opioid agonist therapy. A significant barrier for the staff was the necessity of having a physician present for intakes. Some patients suggested improvements for accessibility, particularly for the disabled or elderly. Overall, the Great Circle MMU enhanced access to opioid agonist therapy for both American Indians/Alaska Natives and non-natives in rural communities. |
| Gibbons [^42^](https://www.zotero.org/google-docs/?jXoxft) 2024 Louisiana | Simulation | Beneficiaries of Louisiana's Medicaid program with at least one claim of diagnosed opioid dependence. | A study predicts that introducing 10 new mobile methadone units in Louisiana would increase the net MOUD (Medication for Opioid Use Disorder) treatment rate by 0.54–2.39 percentage points. Exclusively serving rural areas, these units could boost rural MOUD treatment by 8.54–13.67 percentage points, bringing roughly 20% of rural beneficiaries an average of 24 miles closer to treatment. Mobile methadone units are a promising innovation for increasing methadone use, especially in underserved rural locations, but their impact varies based on operational location, necessitating careful planning to maximize benefits. |
| Fixler[^50^](https://www.zotero.org/google-docs/?XCCLq1) 2024 PA, Pittsburgh | Non-randomised experimental study | Pittsburgh residents in neighborhoods with high overdose data, crime statistics, new HepC and HIV diagnoses, absence of community resources and treatment programs. | The study found a significant decrease in quarterly arrests after treatment was introduced, with a 37.7% reduction in block groups within 1 mile of a clinic and an 18% reduction in more distant areas. After the clinics were established, total arrests dropped by 34.13% and non-drug-related arrests by 22.29% within one mile of the clinics compared to areas further away. There was no significant increase in crime near the clinics, countering concerns that harm reduction services might attract local crime |
| Chatterjee[^38^](https://www.zotero.org/google-docs/?E3Tr5C) 2024 Boston, MA and Ohio | Qualitative research/Interviews | Program staff from mobile MOUD clinics | Organizations leveraged community relationships and interpersonal skills to creatively offer services through mobile MOUD units. Community engagement and partnerships were crucial for their success. The units provided buprenorphine and naltrexone but not methadone due to regulatory barriers. Significant challenges included stigma, limited resources, and financial sustainability. Participants noted that being part of the federally funded HEALing Communities Study facilitated the establishment of mobile units. However, there were barriers at multiple levels, including logistical issues, emergency repairs, and regulatory challenges. Despite these obstacles, mobile MOUD units were seen as innovative solutions to expand access to medications, promote equity in treatment, and reduce stigma. |
| Stewart[^37^](https://www.zotero.org/google-docs/?lpbPws) 2023 Philadelphia, PA | Qualitative research/Interviews | Leadership and staff of mobile OUD care units. | Mobile Opioid Care Units (MOCUs) operated 7 to 35 hours per week and served 20 to over 100 individuals with varying touchpoints per patient. Services include wound care, pregnancy testing, STI testing, HIV and hepatitis C screening, primary care, and naloxone for overdose reversal besides buprenorphine prescriptions. Practical challenges included limited space, patient accessibility, and volume constraints. Funding is a significant barrier, with most units relying on grants and inadequate medical service reimbursements. |
| Wenzel [^20^](https://www.zotero.org/google-docs/?GCJ52o) 2021 Baltimore, Maryland | Case report | Young adults with opioid use disorder (OUD) | Mobile van delivery of extended-release medications for opioid use disorder (XR-MOUD) during the COVID-19 pandermic, offered advantages such as reducing COVID-19 exposure risk, addressing confidentiality concerns, and enhancing security. However, a potential limitation is the lack of in-home settings, which previously allowed family members or treatment significant others to provide immediate persuasion for treatment adherence. |
| Suen[^21^](https://www.zotero.org/google-docs/?m2pgkU) 2023 San Franciso, CA | Qualitative research/Interviews | Providers and patients of mobile opioid treatment programs | The implementation of a methadone van service rapidly during the COVID-19 pandemic faced logistical challenges with medication storage and Wi-Fi connectivity for electronic health records (EHR).Patients appreciated the streamlined efficiency, accessibility, and positive environment at the van improved overall patient experiences. However, counselors encountered difficulties with telehealth counseling, especially for patients without phones, and faced challenges in maintaining consistent contact due to patients' transient presence at the van. |
| Stewart[^54^](https://www.zotero.org/google-docs/?YT66sh) 2021 Philadelphia, PA | Cohort study | Individuals with OUD in neighborhoods with a very high concentration of opioid overdose morltality, server by the Merakey Parkside MEU program. | 11% of individuals accessed Merakey Parkside via a Mobile Engagement Unit (MEU), while 89% came through other referral sources such as walk-ins, a call center, or other treatment providers. MEU patients had lower pre-service utilization of methadone maintenance and outpatient/intensive outpatient substance use treatment services compared to the comparison group. |
| Selitsky[^35^](https://www.zotero.org/google-docs/?mjyb8v) 2022 Baltimore, Maryland | Cohort study | Individuals who use opiates | Higher daily doses of sublingual buprenorphine (>16 mg) were significantly associated with increased odds of patients remaining engaged in treatment for at least 30 days (p < 0.001). Conversely, female patients had lower odds of 30-day retention (AOR = 0.43, 95% CI = 0.19–0.95, p = 0.04), as did those experiencing homelessness (AOR = 0.23, 95% CI = 0.19–0.52, p < 0.001), and those with prior buprenorphine treatment history. |
| Rosenblum[^43^](https://www.zotero.org/google-docs/?xxtIFM) 2002 New York, NY | Non-randomised experimental study | Patients who had at least one encounter with the mobile medical van in 1997. | The van provided Integrated Case Management (ICM), including on-site case management services, further enhanced outcomes by helping individuals access and maintain public assistance benefits. The group with medical encounters provided by the van showed significant reductions in drug use, homelessness, and health complaints among participants. The experimental group showed fewer days of "crack" use (4.1 vs. 2.2 days), fewer days homeless (13.2 vs. 10.2 days), and fewer health complaints (6.3 vs. 4.8) over 30 days compared to controls. |
| Rosecrans[^34^](https://www.zotero.org/google-docs/?YqQB61) 2022 Baltimore, MD | Cohort study | People who inject drugs using the services of The Spot mobile clinic. People who are at risk of overdose and infectious diseases. | The service provided to 569 patients included buprenorphine for 73.8%. Buprenorphine retention rates were 56% at one month and 26.2% at three months, with higher retention rates observed in Black patients and those with hepatitis C. Patients on buprenorphine were more likely to be tested for HIV, hepatitis C, and sexually transmitted infections, and received hepatitis C treatment, vaccinations, naloxone, HIV treatment, and pre-exposure prophylaxis (PrEP). Eight patients out of the nineteen who started treatment for hepatitis C were cured. |
| Regis[^44^](https://www.zotero.org/google-docs/?s8ikFq) 2020 Boston, MA | Qualitative research/Interviews | Vulnerable populations include homeless individuals with high overdose risk. | In its initial 10 months, the program engaged >3,800 individuals who use drugs, resulting in 308 clinical encounters. Services were gradually expanded over time. Among unique patients, prescriptions were provided, and 28 individuals underwent follow-up toxicology screenings, revealing positive results for buprenorphine in some cases and negative results for opioids in others. Qualitative interviews indicated that the model was highly acceptable to individuals experiencing stigma in traditional healthcare settings, emphasizing the value of compassionate and flexible care. Participants expressed a strong demand for medical services combined with proactive street outreach, highlighting convenience as a major benefit of the model. |
| Pepin[^27^](https://www.zotero.org/google-docs/?t5ctOw) 2023 Worcester, MA | Qualitative research | Patients with opioid use disorders who are experiencing homelessness | Nearly half of patients accessed the community-based mobile addiction service only once, while the other half had repeat visits. Buprenorphine/naloxone (Suboxone) was prescribed to 29.4% of all patients, with a significant portion receiving multiple visits for ongoing care.  One of the major challenges highlighted was managing patient care disruptions caused by forced removals of encampments by city authorities, which resulted in the loss of patient belongings and medications. |
| O'Gurek[^26^](https://www.zotero.org/google-docs/?5vkQj9) 2021 Philadelphia, PA | Cohort study | Individuals experiencing homelessness and opioid use disorder | The study focused on a population predominantly experiencing homelessness (90%), intravenous opioid use (67%), and prior treatment for substance use disorder (92%). Retention rates in care were observed at 61.2% at 1 month, 36.6% at 3 months, and 27.6% at 5 months. Barriers such as the transient nature of homelessness likely contributed to lower retention rates, impacting continuous maintenance with buprenorphine. |
| Nuttbrock[^22^](https://www.zotero.org/google-docs/?sr7mIn) 2003 New York City, NY | Cohort study | Patients experiencing homelessness in New York City | The study focused on a mobile medical van providing services to homeless individuals with SUDs. Among the clients, high percentages used cocaine (76.9%) and opiates (16.5%) within the past month, with 48.2% assessed as drug dependent. Medical follow-up was scheduled for 90% of clients, and medications were prescribed or dispensed in 28% of visits. Referrals to other healthcare providers were infrequent. A significant portion tested positive for HIV (21.4%), hepatitis B exposure (47.8%), hepatitis C (36.5%), syphilis (11.5%), and tuberculosis (46.9%). Many clients also showed depressive symptoms (38.8%) and psychotic symptoms (15.9%). |
| Noyes[^19^](https://www.zotero.org/google-docs/?5tzLnj) 2021 Boston, MA | Qualitative research/Interviews | Adolescents and young adults who use drugs and are at a higher risk of overdose. | During the COVID-19 pandemic, the CCIR mobile van distributed syringes and naloxone. The pandemic disrupted services, causing missed contacts and reduced distributions due to logistical and safety concerns. Harm reduction programs faced staffing shortages, reduced hours, and difficulties in securing supplies like naloxone and syringes. People who use drugs (PWUD) encountered increased risks of overdose, withdrawal, and infection due to reduced access to resources and altered living conditions during the pandemic. Policy barriers, such as parental consent requirements for adolescents seeking treatment, also hindered access to care. |
| Messmer[^55^](https://www.zotero.org/google-docs/?5YZ6Br) 2023 Chicago, IL | Cohort study | Patients in traditionally medically un­derserved Chicago neighborhoods with the highest overdose rates | A mobile medical unit focused on primary care and opioid use disorder (OUD) treatment served nearly 600 unique patients, with over 200 seeking buprenorphine initiation or follow-up. The unit responded to community overdoses and saw a high proportion of African American patients (64.6%). Buprenorphine treatment was a significant reason for 51.6% of follow-up visits, with some patients preferring to continue care at the mobile unit rather than traditional healthcare facilities. Data collection was limited to electronic medical records (EMR), affecting consistency in documenting substance use patterns. The program emphasizes a holistic approach, integrating harm reduction and medical treatment to enhance patient trust and engagement, particularly in utilizing Medications for Opioid Use Disorder (MOUD). |
| Lowenstein[^36^](https://www.zotero.org/google-docs/?L4XQgF) 2023 Philadelphia, PA | Qualitative research/Interviews | Patients of low-barrier buprenorphine program | Participants in the program highlighted positive interactions with staff and care providers as crucial for their engagement. Respectful and encouraging interactions fostered connections and contributed to treatment success. Some participants at busier sites experienced long waits or had to return due to limited spots, suggesting a need for more staff to address these issues. Street-based care posed challenges such as inclement weather, lack of privacy, and discomfort due to lack of anonymity while waiting outdoors. Participants sought more comprehensive mental health support not always available on-site, facing difficulties accessing these services elsewhere.  The low-barrier model of the program was praised for its convenience, flexibility, and the supportive, empathetic care team, contrasting positively with participants' past treatment experiences. Robust case management and peer support were also highlighted as beneficial aspects of the program, addressing social determinants of health and enhancing patient satisfaction. |
| Langabeer[^32^](https://www.zotero.org/google-docs/?S3RJg5) 2020 Houston, TX | Cross sectional study | Patients at the ED of Memorial Hermann Hospital (MHH) with OUD and lack of current enrollment in OUD treatment. | Out of 103 individuals identified for the study, 69 chose not to participate in interviews. Despite challenges, the treatment program showed promising retention rates with 88% of participants remaining after 30 days and 56% after 90 days. Three patients who relapsed continued in the program. |
| Kuo[^56^](https://www.zotero.org/google-docs/?wcMiXC) 2003 Baltimore, Maryland | Non-randomised experimental study | Attenders of a Needle exchange program (NEP) in Baltimore, MD. | Nearly two-thirds of individuals entering the mobile LAAM program had never been enrolled in a non-detoxification drug treatment program before. Participants in the program showed significant reductions in drug, alcohol, and legal issues as measured by the ASI scores after one month compared to non-participants, ( p < .0001),( p = .003), ( p = .004) respectively. Specifically, there was a 31% decrease in opiate-positive urine tests and a 22% decrease in cocaine-positive tests between the first and third months of treatment |
| Krawczyk[^29^](https://www.zotero.org/google-docs/?LjGEPh) 2019 Baltimore, MD. | Cohort study | Opioid-dependent patients with justice involvement and psychosocial vulnerabilities, initiating buprenorphine treatment. | The program focused on initiating buprenorphine treatment for a demographic primarily consisting of African American males, with a mean age of 44.1 years and an average opioid use duration of 24.0 years. The majority had previous criminal justice involvement (94.4%), were unemployed (72.9%), and were unstably housed (70.8%). Over a third had a history of overdose (32.1%). The program facilitated agreements with pharmacies for billing non-profits in cases of lack of insurance, provided services for obtaining identification, and allowed for same-day visit and buprenorphine initiation, enhancing accessibility. Nearly 68% of patients returned for subsequent visits, and 31.6% remained in treatment after 30 days. About 20.5% were transferred to continue buprenorphine treatment at partnering sites. |
| Komaromy[^18^](https://www.zotero.org/google-docs/?rpfbFX) 2021 Boston, MA | Text and opinion | Adolescents abd young with substance use disorders (SUDs) during the COVID-19 pandemic. | CATALYST Program: Referrals increased significantly in March, and visit show-rates improved from approximately 60% to 70% after transitioning to telehealth for adolescents and young adults.  Project TRUST: Despite decreased physical access to treatment services in 2020 due to the pandemic, medical visits for Substance Use Disorders (SUD) remained consistent with 2019 levels, totaling 282 visits over a 6-week period.  Faster Paths: Similarly, the number of visits remained stable at 282 in 2020 compared to 298 in 2019 during the same 6-week period, despite reduced physical access to treatment services.  ABOVE Program: This program, focusing on patients with co-occurring SUDs and mental illness, found that most patients preferred telehealth visits. However, those with serious mental illness required scheduled face-to-face appointments for adequate assessments and engagement, including regular urine testing and drop-in appointments.  BMC’s SUD Treatment Programs: During the COVID-19 pandemic, BMC's SUD treatment programs adapted by shifting most service delivery to telehealth. They tailored these adaptations to meet the diverse needs of their patient population, establishing new programs like the COVID Recuperation Unit and adjusting existing programs such as mobile outreach for mother-child dyads affected by SUDs. These efforts enabled continued provision of harm reduction and SUD treatment services despite the challenges posed by the pandemi |
| Iheanacho[^24^](https://www.zotero.org/google-docs/?yV9fMx) 2020 New Haven, CT | Retrospective chart review | Veterans experiencing homelessness | The team uses a mobile video conferencing app to facilitate psychiatric evaluations and initiate buprenorphine (BUP) treatment, addressing barriers such as transportation and missed appointments associated with clinic-based care.  Retention rates in the M-CAT program ranged from 100% at 1 month to 75% at 12 months, with an average treatment duration of 19.2 months. While retention rates were lower compared to the traditional BUP clinic (100% retention at endpoint), they were higher than similar low-barrier interventions for veterans and non-veterans with OUD who are homeless. Mean retention in treatment was 19.2 months (standard deviation [SD] = 10.2) in M‐CAT and 36 months  (SD = 27.6) in BUP clinic. At the endpoint, 66.7% (n = 8) in M‐CAT and 100% (n = 24) in BUP clinic remained on BUP. |
| Harris[^33^](https://www.zotero.org/google-docs/?wlhc9f) 2022 Baltimore, MD | Cross sectional study | Patients of The Spot, a mobile street medicine program in Baltimore. | The Spot transitioned 176 active patients from in-person opioid use disorder (OUD) treatment to telemedicine services. During the transition, 129 patients (73.2%) continued engagement through telemedicine, while 47 (26.7%) were lost to follow-up. Two previously inactive patients were re-engaged via telemedicine. Demographic analysis showed no significant differences in age, gender, race, or ethnicity between patients who transitioned and those lost to follow-up. Retention rates were high with 80.7% of patients actively engaged in OUD treatment at the study's end, 16.0% lost to follow-up, and 3.3% deceased. Telemedicine engagement was supported by flexible service delivery and extended prescription lengths, contributing to high retention rates comparable to in-person services. |
| Hall[^57^](https://www.zotero.org/google-docs/?yN3uZn) 2014 New Jersey | Cross sectional study | Individuals enrolled in NJ-MATI program | NJ-MATI (New Jersey Medication-Assisted Treatment Initiative) clients, often referred through street outreach and syringe exchange programs, were more likely to be African American, homeless, and uninsured compared to traditional methadone clients. Public funding eliminated affordability as a barrier for treatment participation. Services offered six days a week included methadone maintenance, Suboxone detoxification, induction, maintenance, and office-based care. Clients received vouchers for additional treatment and support services.  NJ-MATI clients had higher rates of co-occurring mental illness (OR = 1.6, 95% CI = 1.3, 2.1) compared to traditional methadone clients but showed no significant difference in court supervision. |
| Grieb[^58^](https://www.zotero.org/google-docs/?KJWTmo) 2022 Baltimore, MD | Qualitative research/Interviews | PWUD accessing mobile health clinic partnered with syringe services program. | People who use drugs viewed a mobile health clinic in their neighborhood positively, seeing benefits such as improved access to healthcare services, low-threshold buprenorphine dispensation, and services without drug-use stigma. Word-of-mouth was the primary way they learned about the clinic, which limited their access due to incomplete information about the services provided. Non-clients typically used methadone instead of buprenorphine. |
| Greenfield[^31^](https://www.zotero.org/google-docs/?VOcX1p) 1996 Baltimore, MD | Cross sectional study | Patients admitted to the Mobile health service in neighborhoods selected had high rates of drug use. | Only 12.8% of mobile program patients were referred by service agencies, compared to 28.7% in fixed-site programs (P < 0.001). Mobile program patients exhibited a median retention of 15.53 months, significantly higher than the 3.90 months for fixed-site patients in MHS served zip codes (MHSZIPS) and 6.27 months for those from other zip codes (P < 0.001). Methadone doses averaged 60 mg in the mobile program and ranged from 50-65 mg in fixed-site programs. Predictors of shorter retention included a higher number of arrests, more frequent cocaine use, and lower family income, with these factors being more prevalent among MHSZIPS patients. Additionally, MHSZIPS patients were more likely to use cocaine daily, have an income below $5000, and depend on public assistance compared to patients from other zip codes. Overall, mobile program patients demonstrated significantly longer retention compared to those in fixed-site programs. |
| Gibson[^51^](https://www.zotero.org/google-docs/?LcEfox) 2017 New Haven, CT | Cohort study | Patients from medically marginalized communities served by the The New Haven Community Health Care Van. | CHCV (Community Health Care Van) delivers services directly to impoverished and underserved communities in New Haven. From January 2004 to December 2012, 29,728 visits were recorded, with latent tuberculosis screening being the most frequent service (24.5%). Mental health services accounted for 11.4% of visits, primarily provided as ancillary counseling for patients on buprenorphine maintenance therapy. Key factors leading to higher healthcare utilization included being foreign-born, injecting drugs, having hypertension, and being HIV-infected, while fewer visits were associated with not completing high school and engaging in sex work. The program utilized Spanish interpreter services allowing to care for LEP. |
| Gibson[^59^](https://www.zotero.org/google-docs/?0rudtz) 2014 New Haven, CT | Cross sectional study | Patients of the community healthcare van (CHCV) | MMCs (Mobile Medical Clinics) attract clients despite not being geographically close to SAMHSA facilities. Participants living both within five miles and more than fifty miles of MMCs tend to be less stably housed and uninsured, with those traveling over fifty miles significantly more likely to be homeless (40.3%, p=0.03). High-frequency users of MMCs are predominantly men (66.7%, p<0.01), almost all foreign-born (94.4%, p<0.01), with about 15% traveling more than five miles for care. Over half are unstably housed, 85% unemployed, and they report higher rates of drug use, injecting drugs, recent incarceration, sex solicitation, sex work, domestic violence, and sexual assault. These clients also have higher rates of health conditions, including HIV infection, with over half receiving substance use treatment via medication-assisted therapy and 75% being HIV-infected. The ACA lacks provisions for MMCs, which serve vulnerable populations with stigmatized conditions. Factors beyond geographic proximity and lack of health insurance motivate clients to seek continuous care at MMCs designed for acute, episodic care and linkage to other services. |
| Fine[^25^](https://www.zotero.org/google-docs/?Mw9eg8) 2021 Massachusetts | Qualitative research/Interviews | Patients experiencing homelessness receiving clinical care from a mobile addiction-focused outreach program. | The mobile health program attracted individuals primarily for specific services such as new needles (33.0%), food/drink (27.5%), and buprenorphine prescriptions (25.3%), though respondents ended up using a median of five services provided by the program. Almost all respondents trusted and felt respected by the program staff (98.9%) and believed the program met their healthcare needs (97.8%). Additionally, 70% reported they would decrease their drug or alcohol use because of the program, and all participants would recommend it to their friends. The most frequently suggested improvements were adding behavioral health services, hepatitis C treatment, and more information about program hours and locations. The mobile program was preferred over traditional office-based care, especially regarding wait times, treatment of addiction, and homelessness. Prior to the program, many had limited healthcare access, with 20.9% not seeing a provider for over three years and 62% reporting past unfair treatment due to their housing status, substance use, or inability to pay. |
| Fine[^45^](https://www.zotero.org/google-docs/?stLUSz) 2023 Boston, MA | Non-randomised quasi-experimental study | Adults who engaged with the mobile addiction program run by Boston  Health Care for the Homeless Program | In a cohort of people experiencing homelessness, the mobile addiction program saw a non-significant increase in the yearly mean number of outpatient visits from 11.5 to 12.1 (p=0.42), while the control cohort experienced a decrease from 12.4 to 9.9 (p=0.12). Inpatient hospitalizations for the mobile program cohort increased significantly from 2.2 to 3.0 (p=0.04), but remained stable at 2.5 in the control cohort (p=0.82). Overall, the mobile addiction program did not significantly affect healthcare utilization compared to a large Health Care for the Homeless program in Boston, MA. |
| Busen[^23^](https://www.zotero.org/google-docs/?OneY2r) 2008 Houston, Texas | Cohort study | Youth aged 15-25 experiencing homelessness utilizing a mobile medical unit | The mobile unit program for homeless youth provided psychiatric interventions primarily through counseling (56.8%), medication stabilization (43%), and hospitalization (9%). Over an average visit duration of 14 months, 52% of males and 74.5% of females showed improvement. The sample, which had multiple psychiatric disturbances (76%) and high rates of substance use (46.8%), often reported polysubstance use and histories of suicidal behavior (23%) linked to depression from use, molestation, and abandonment. The program effectively improved self-worth and mood stabilization and was successful in screening and treating STIs, as well as viral infections like hepatitis, HIV, and HPV. The findings were consistent with other homeless youth samples, highlighting the importance of comprehensive mental and physical health services for this vulnerable population. |
| Bowser[^28^](https://www.zotero.org/google-docs/?Bsmv4V) 2010 Oakland, California | Cohort study | Men and women who are on criminal probation and who have histories of drug use | Clients reported reductions in alcohol, crack, cocaine, and heroin use, and a decrease in the number of crimes committed. Despite an increase in days spent in jail, injection drug use declined. Participants experienced reduced stress from drug use and improved self-rated health, with a higher percentage of program completers reporting better health outcomes. Employment rates also increased from 13% at intake to 31% at 12 months. Program completers, who engaged more frequently with the services, showed significantly better outcomes compared to non-completers. |
| Bartholomew[^60^](https://www.zotero.org/google-docs/?MaB3nV) 2022 Florida, Miami-Dade County | Semi-structured qualitative interview and quantitative demographic assessment. | Black patients who inject drugs | Participants felt that the SSP mobile unit is more accessible than other SUD treatment and HIV prevention programs, and suggested that its accessibility could be further improved through strategic locations and extended hours. They believed that delivering buprenorphine treatment via the mobile unit would be accepted and feasible, and that the mobile unit would effectively address barriers related to transportation, cost, and stigma that are common in traditional healthcare settings. Participants also felt that the mobile SSP would be a safe, confidential, and comfortable location for receiving medications for opioid use disorder (MOUD) and PrEP. They emphasized the importance of having caring, nonjudgmental, approachable staff with lived drug use experience. |
